# Clinical, Bronchoscopic, and Imaging Findings of e-Cigarette, or Vaping, Product Use–Associated Lung Injury Among Patients Treated at an Academic Medical Center

**DOI:** 10.1001/jamanetworkopen.2020.19176

**Published:** 2020-11-06

**Authors:** Scott K. Aberegg, Meghan M. Cirulis, Sean D. Maddock, Andrew Freeman, Lynn M. Keenan, Cheryl S. Pirozzi, Sanjeev M. Raman, Joyce Schroeder, Howard Mann, Sean J. Callahan

**Affiliations:** 1 Division of Pulmonary and Critical Care Medicine, University of Utah Health, Salt Lake City; 2 Division of Respiratory, Critical Care, and Occupational Pulmonary Medicine, University of Utah School of Medicine, Salt Lake City; 3 George E. Wahlen Department of Veterans Affairs Medical Center, Salt Lake City, Utah; 4 Department of Radiology and Imaging Sciences, University of Utah, Salt Lake City

## Abstract

**Importance:**

e-Cigarette, or vaping, product use–associated lung injury (EVALI) has caused more than 2800 illnesses and 68 deaths in the United States. Better characterization of this novel illness is needed to inform diagnosis and management.

**Objective:**

To describe the clinical features, bronchoscopic findings, imaging patterns, and outcomes of EVALI.

**Design, Setting, and Participants:**

This case series of 31 adult patients diagnosed with EVALI between June 24 and December 10, 2019, took place at an academic medical center in Salt Lake City, Utah.

**Exposures:**

e-Cigarette use, also known as vaping.

**Main Outcomes and Measures:**

Symptoms, laboratory findings, bronchoscopic results, imaging patterns, and clinical outcomes.

**Results:**

Data from 31 patients (median [interquartile range] age, 24 [21-31] years) were included in the study. Patients were primarily men (24 [77%]) and White individuals (27 [87%]) who used e-cigarette products containing tetrahydrocannabinol (THC) (29 [94%]). Patients presented with respiratory (30 [97%]), constitutional (28 [90%]), and gastrointestinal (28 [90%]) symptoms. Serum inflammatory markers were elevated in all patients. Bronchoscopy was performed in 23 of 28 inpatients (82%) and bronchoalveolar lavage (BAL) revealed the presence of lipid-laden macrophages (LLMs) in 22 of 24 cases (91%). BAL samples tested positive for *Pneumocystis jirovecii* (3 patients [13%]), rhinovirus (2 patients [8%]), human metapneumovirus and *Aspergillus* (1 patient each [4%]); all except human metapneumovirus were determined to be false-positives or clinically inconsequential. The exclusive or dominant computed tomography (CT) pattern was organizing pneumonia in 23 of 26 cases (89%). Patients received antibiotics (26 [84%]) and corticosteroids (24 [77%]), and all survived; 20 patients (65%) seen in follow-up showed marked improvement, but residual symptoms (13 [65%]), radiographic opacities (8 [40%]), and abnormal pulmonary function tests (8 of 18 [44%]) were common.

**Conclusions and Relevance:**

In this case series, patients with EVALI characteristically presented with a flu-like illness with elevated inflammatory markers, LLMs on BAL samples, and an organizing pneumonia pattern on CT imaging. Bronchoscopic testing for infection had a high incidence of false-positive results. Patients had substantial residual abnormal results at early follow-up. These data suggest a limited role for bronchoscopy in typical presentations of EVALI without risk factors for alternative diagnoses and the need for careful longitudinal follow-up.

## Key Points

**Question:**

What are the typical clinical, radiographic, and bronchoscopic findings and clinical outcomes of e-cigarette, or vaping, product use–associated lung injury (EVALI)?

**Findings:**

This case series of 31 patients found that EVALI typically presented as a flu-like illness with elevated inflammatory markers and an organizing pneumonia pattern on computed tomography imaging. Bronchoscopy showed lipid-laden macrophages and had a high rate of false-positive results for infection.

**Meaning:**

The findings of this study suggest that EVALI has a characteristic clinical and radiographic presentation and that bronchoscopy has limited utility in its evaluation.

## Teaser

This case series describes the clinical features, bronchoscopic findings, imaging patterns, and outcomes of e-cigarette, or vaping, product use–associated lung injury (EVALI).

## Introduction

In early 2019, a flu-like illness associated with the use of e-cigarettes was identified in the United States.^1,2,3,4,5^ Since that time, more than 2800 cases of e-cigarette, or vaping, product use–associated lung injury (EVALI) have been reported to the US Centers for Disease Control and Prevention (CDC), resulting in at least 68 deaths.^6,7,8,9,10,11^ Our understanding of this syndrome is based on 4 large patient series, which together comprised approximately 315 patients, less detailed CDC data, and some smaller case series.^1,8,12,13,14,15,16,17,18,19,20^ Despite these reports, numerous uncertainties remain, including information on bronchoscopic findings, characteristic radiographic patterns, and longitudinal outcomes.

Utah has experienced among the highest per capita EVALI rates in the United States.^10,21^ The University of Utah Medical Center (UUMC) is a quaternary care academic medical center in Salt Lake City, Utah, serving a large referral area. We identified a case of EVALI at UUMC during the final week of June 2019. Bronchoalveolar lavage (BAL) specimens from this and subsequent patients revealed the presence of lipid-laden microphages (LLMs), which led to suspicion the illness was a form of exogenous lipoid pneumonia.^2,22^ We now know that this is probably inaccurate,^13,23,24,25^ but it prompted a local practice of routine bronchoscopy for patients suspected of having EVALI. As a result, most of the first 31 patients seen at UUMC underwent bronchoscopy with extensive molecular and microbiological testing during the first 5 months of the outbreak.

In this article, we describe the presentation, evaluation, and clinical course of patients diagnosed with EVALI at UUMC. A high rate of bronchoscopy, detailed characterization of imaging findings by 2 thoracic radiologists, and follow-up data for most patients resolve some of the remaining uncertainties regarding EVALI and provide a more complete picture of a typical case than has, to our knowledge, been reported.

## Methods

The initial 6 cases of EVALI identified at UUMC were cared for by 1 of the authors (S.K.A., M.M.C., S.D.M., L.M.K. C.S.P., S.M.R, or S.J.C.) between June 24 and August 16, 2019, and have been previously reported^2^; they are included in this analysis as well. Thereafter, as the magnitude of the outbreak became apparent, institutional emails and informal communications encouraged clinicians to admit patients with suspected EVALI to a service staffed by the pulmonary faculty (pulmonary inpatient or medical intensive care unit [ICU] services, as appropriate) or to contact the pulmonary services for consultation and arranging outpatient follow-up. An ad hoc vaping clinic was created within the general pulmonary clinic to facilitate follow-up after discharge and to accommodate outpatient referrals. In addition, 2 of us (S.K.A. and S.J.C.) acted as liaisons between UUMC and the Utah Department of Health to report cases and facilitate exchange of information. These processes assured that few, if any, patients with EVALI were not identified for this report. Only cases reviewed by 2 of us (S.K.A. and .S.J.C.) and determined to be a confirmed or probable diagnosis of EVALI based on the CDC case definition (ie, e-cigarette use within 90 days of symptom onset, radiographic infiltrates, a negative preliminary infectious workup, and no other plausible diagnoses) were included.^18^ We stopped including patients when the current analysis was begun on December 10, 2019. Internal review board approval was granted for this case series, and informed consent was waived because the data were deidentified. This study followed the the reporting guideline for case series.

One of us (S.J.C.) reviewed each patient’s medical record and abstracted data for presented variables. Other authors (M.M.C., S.D.M., L.M.K., S.M.R., A.F., and C.S.P.) reviewed data for each patient to ensure accuracy. Bronchoscopic data were manually extracted for the following variables: bronchoalveolar fluid cellular count and differential, respiratory virus polymerase chain reaction (PCR), bacterial and fungal stains and cultures, *Aspergillus galactomannan* antigen, *Legionella* by qualitative PCR, *Chlamydia pneumoniae* by PCR, and *Pneumocystis jirovecii* by PCR. Oil red-O stains were performed on all but 1 BAL; results represent the cytopathologist’s quantification of the percentage of LLMs observed in each sample according to established local laboratory practices.^26^

Two thoracic radiologists (H.M. and J.S.) jointly and retrospectively reviewed chest radiographs and computed tomography (CT) images, classifying (by informal consensus) the latter into 1 or more of the following previously reported patterns: organizing pneumonia (OP), diffuse alveolar hemorrhage (DAH), acute lung injury (ALI), acute eosinophilic pneumonia (AEP), hypersensitivity pneumonitis (HP), and exogenous lipoid pneumonia (ELP).^3,26^ When applicable, a dominant and minor pattern were recorded. Images were also assessed for the presence of airway wall thickening in relation to segmental bronchi.^27^

### Statistical Analysis

Summary and descriptive statistics were calculated using Stata version 15.1 (StataCorp). No prespecified level of statistical significance was set.

## Results

### Baseline Characteristics

Table 1 shows the demographic characteristics, vaping exposures, and clinical characteristics of the 31 patients. Most were White individuals (27 [87%]) and men (24 [77%]), with a median (interquartile range [IQR]) age of 24 (21-31) years. Most (29 [94%]) reported vaping tetrahydrocannabinol (THC) either alone (12 [39%]) or in combination or alternation with nicotine (17 [55%]). Frequency of THC vaping was difficult to quantify due to a number of factors, including variable qualities of vaping histories obtained, patients omitting or underreporting actual usage due to the use of illicit substances, and a range of usage frequencies reported, such as a few times per day to almost continuous use. Few patients reported changes in the frequency of their vaping temporally related to their illness; however, many did report subjective changes they noticed in the quality of the products they were using, be it taste or even visual assessment of the viscosity of the liquid. All patients presented with at least 1 symptom from the following categories: respiratory (30 [97%]), constitutional (28 [90%]), or gastrointestinal (28 [90%]); 24 (77%) had symptoms from all these categories. The most common individual symptoms within each category were dyspnea (30 [97%]) and cough (25 [81%]); fever (28 [90%]) and chills (20 [65%]); and nausea (27 [87%]) and vomiting (24 [77%]).

**Table 1.** Demographic Characteristics and Presenting Symptoms

Characteristic	Patients, No. (%) (N = 31)^a^
Age, median (IQR), y	24 (21-31)
Men	24 (77)
Ethnicity	
Not Hispanic or Latino	24 (77)
Hispanic or Latino	7 (23)
Race	
White	27 (87)
Native Hawaiian or other Pacific Islander	1 (3)
Unknown or not reported	3 (10)
Tobacco use	
Current	8 (26)
Former	9 (29)
Never	14 (45)
Cigarette packs per day, median (IQR)	0.2 (0.1-1.0)
Years smoked, median (IQR)^b^	4 (2-15)
Type of e-cigarette	
Nicotine	2 (7)
Marijuana or THC	12 (39)
THC and nicotine	17 (55)
No THC or nicotine	0
Time since starting vaping, median (IQR), mo	12 (3-24)
Presenting symptoms	
Any constitutional, respiratory, or gastrointestinal symptom	31 (100)
≥1 constitutional, respiratory, and gastrointestinal symptom	24 (77)
Any constitutional symptom	28 (90)
Fever	28 (90)
Chills	20 (65)
Night sweats	13 (42)
Any respiratory symptom	30 (97)
Cough	25 (81)
Shortness of breath	30 (97)
Chest pain	17 (55)
Any gastrointestinal symptom	28 (90)
Nausea	27 (87)
Vomiting	24 (77)
Abdominal pain	10 (32)
Diarrhea	10 (32)

Abbreviations: IQR, interquartile range; THC, tetrahydrocannabinol.

^a^ Percentages may not sum to 100 due to rounding.

^b^ Data on years smoked available for 8 patients.

### Laboratory Findings

Laboratory and bronchoscopic findings are summarized in Table 2. Most patients (28 [90%]) had a mild peripheral leukocytosis with a median (IQR) white blood cell count of 15 300/μL (12 300/μL-17 900/μL [to convert to ×10^9^ per microliter, multiply by 0.001]) and a granulocyte predominance (median [IQR] granulocyte percentage, 90.3% [88.0%-92.4%]). The erythrocyte sedimentation rate and C-reactive protein levels were elevated in 25 of 26 patients (96%) and 31 patients (100%), respectively, with median (IQR) values of 75 (42-100) mm/h and 25.8 (18.7-30.2) mg/dL (to convert to milligrams per liter, multiply by 10), respectively. Overall, 7 of 26 patients (27%) had erythrocyte sedimentation rate values of at least 100 mm/h. A minority had mild elevations in transaminases, and hematuria was noted in 8 of 21 urinalysis samples (38%). HIV serologies were negative in all 19 patients for whom this was determined.

**Table 2.** Laboratory Studies on Initial Presentation

Laboratory study	Median (IQR)
White blood cell count, /μL	15 300 (12 300-17 900)
Differential, median (IQR), %	
Granulocytes	90.3 (88.0-92.4)
Lymphocytes	5.7 (4.2-7.9)
Monocytes	2.4 (2-3.2)
Basophils	0.2 (0.1-0.3)
Eosinophils	0.3 (0.1-0.9)
ESR, mm/h	75 (42-100)
CRP, mg/dL	25.8 (18.7-30.2)
Elevated, No./total No. (%)	
ESR^a^	25/26 (96)
CRP^b^	27/27 (100)
ESR or CRP	28/28 (100)
ESR >100 mm/h	7/26 (27)
Procalcitonin, ng/mL	0.3 (0.1-0.7)
Creatinine, mg/dL	0.85 (0.73-0.94)
Total bilirubin, mg/dL	1.0 (0.6-1.4)
AST, U/L	31 (25-37)
ALT, U/L	24 (18-39)
Elevated, No./total No. (%)	
AST^c^	6/30 (20)
ALT^d^	5/30 (17)
AST or ALT	7/30 (23)
Alkaline phosphatase, U/L	85 (72-114)
HIV 1, 2 antigen or antibody	
Negative, No./total No. (%)	19/19 (100)
BAL performed, No./total No. (%)	24/31 (77)
Cytologic differential, %	
Macrophages	53 (33-79)
Neutrophils	28 (12-48)
Lymphocytes	6 (2-12)
Eosinophils	0 (0-2)
Presence of LLMs, No./total No. (%)^e^	21/23 (91)
LLMs, median (IQR), %	52 (33-76)
Urine drug screen, No./total No. (%)	
Marijuana	11/11 (100)
Cocaine	0/11
Heroin	0/1
Methamphetamine	1/11 (9)
Narcotics	2/11 (18)
Benzodiazepine	0/11
Methadone	0/11
Buprenorphine	0/11

Abbreviations: ALT, alanine aminotransferase; AST, aspartate aminotransferase; BAL, bronchoalveolar lavage; CRP, C-reactive protein; ESR, erythrocyte sedimentation rate; IQR, interquartile range; LLM, lipid-laden microphage.

SI conversion factors: To convert AST, ALT, and alkaline phosphatase to microkatals per liter, multiply by 0.0167; creatinine to micromoles per liter, multiply by 76.25; CRP to milligrams per liter, multiply by 10; total bilirubin to micromoles per liter, multiply by 17.104; and white blood cell count to ×10^9^/μL, multiply by 0.001.

^a^ Greater than upper reference limit (20 mm/h).

^b^ Greater than upper reference limit (0.8 mg/dL).

^c^ Greater than upper reference limit (40 U/L).

^d^ Greater than upper reference limit (60 U/L).

^e^ One patient who underwent BAL did not have LLM testing performed.

### Bronchoscopic Features

Among all 31 patients, 24 (77%) underwent bronchoscopy with BAL. Three of the last 4 inpatients (75%) in the series did not undergo bronchoscopy because by mid-November we had stopped performing routine bronchoscopy; the final patient had bronchoscopy because of recurrent symptoms resulting in readmission. The cytologic differential from BAL fluid showed a predominance of macrophages (median [IQR], 53% [33%-79%]) followed by neutrophils (median [IQR], 28% [12%-48%]). Oil red-O staining was performed on 23 samples, and LLMs were present in 21 patients (91%). The median (IQR) proportion of macrophages that stained positive with Oil red-O was 52% (33%-76%).

### Evaluation for Infection

Testing of BAL and noninvasively obtained samples for evidence of infection revealed 1 case (4%) of human metapneumovirus, which was thought to be coincident with EVALI.^28,29,30^ This patient had a prolonged course, with a hospital stay of 21 days, including 14 days in the ICU receiving oxygen via high flow nasal cannula. Two patients (8%) had rhinovirus on PCR testing of BAL fluid.^28,29,31^ Three samples (13%) had positive PCR assays for *Pneumocystis jirovecii* that were considered false-positives because *Pneumocystis* direct fluorescence antibodies were negative and the patients recovered without treatment for *Pneumocystis*. One BAL culture (4%) had growth of *Aspergillus nidulans*, which was deemed a commensal organism or contaminant and not treated; this patient also recovered. Finally, 1 patient (4%) had intermediate positive acute serologies (immunoglobin M) for *Mycoplasma pneumoniae*. In addition to corticosteroids, he was treated with ceftriaxone and azithromycin, but they were discontinued after 2 days when the diagnosis of EVALI was made, and he recovered.

### Imaging

Chest radiography revealed multifocal, multilobar opacities, variable in extent and distribution, consistent with foci of alveolar consolidation. Twenty-six patients (84%) underwent CT scanning (Table 3). eFigure 1 in the Supplement shows examples of the major patterns seen on CT imaging. The OP pattern was the sole pattern in 18 examinations (69%) and the dominant pattern in 5 (19%). The HP pattern was the dominant pattern in 2 examinations (8%). The AEP pattern was dominant in 1 examination (4%). The ELP pattern was nondominant in 1 examination (4%), and the ALI pattern was nondominant in 1 examination (4%). No examinations manifested the DAH pattern. Table 4 summarizes major characteristics of each pattern. Subpleural sparing was present in 15 examinations (58%). Airway wall thickening (eFigure 2 in the Supplement) was present in 21 patients (81%).

**Table 3.** Computed Tomography Findings

Pattern	Patients, No. (%) (n = 26)^a^
Organizing pneumonia^b^	26 (100)
Pneumonitis	
Hypersensitivity^c^	5 (19)
Acute	
Eosinophilic^d^	1 (4)
Lung injury^e^	1 (4)
Exogenous lipoid pneumonia^e^	1 (4)
Diffuse alveolar hemorrhage	0
Subpleural sparing	
Yes	10 (39)
Some	5 (19)
Any^f^	15 (58)
No	11 (42)
Airway wall thickening	
Yes	21 (81)
No	5 (19)

^a^ Percentages may not sum to 100 due to rounding. Totals and percentages for all patterns sum to greater than 26 and 100%, respectively, because some examinations were classified as having more than 1 pattern.

^b^ Organizing pneumonia was the sole pattern in 18 examinations and the dominant pattern in 5 examinations.

^c^ Hypersensitivity pneumonitis was the dominant pattern in 2 examinations.

^d^ Dominant pattern.

^e^ Nondominant pattern.

^f^ Any subpleural sparing includes patients in the yes and some categories.

**Table 4.** Computed Tomography Classification Scheme Used to Establish Joint Consensus by the 2 Radiologists

Pattern^a^	Method of classification
Organizing pneumonia	Peripheral or perilobular patchy bilateral GGOs or consolidation; reverse halo sign; atoll sign
Hypersensitivity pneumonitis	Upper-lung or midlung predominant GGOs; centrilobular nodules; air trapping
Acute eosinophilic pneumonia	Bilateral and symmetric GGOs or consolidation; pleural effusions; septal thickening
Acute lung injury	Acute phase: heterogenous consolidation; GGOs; crazy-paving dependent distribution; organizing phase: development of reticulation and traction bronchiectasis
Diffuse alveolar hemorrhage	Centrilobular nodules; GGOs; consolidation; subpleural sparing
Exogenous lipoid pneumonia	Dependent distribution; GGOs; consolidation; crazy-paving; macroscopic fat attenuation, ≤30 HU
Giant cell interstitial pneumonia	GGOs; architectural distortion; peribronchiolar linear opacities
Airway wall thickening	Qualitative visual analysis

Abbreviations: GGOs, ground glass opacities; HU, Hounsfield units.

^a^ Adapted from Henry TS et al, 2019.^26^

### Management

Most patients (28 [90%]) were admitted to the hospital, and 8 (268%) were treated in the ICU (Table 5). The median (IQR) hospital stay was 4 (3-7) days, with a range of 1 to 21 days, highlighting the variability in illness severity and response to treatment. Most patients (27 [87%]) required supplemental oxygen: 5 patients (16%) required supplemental oxygen delivered by high flow nasal cannula, 3 (10%) required mechanical ventilation, and 1 (3%) was supported with venovenous extracorporeal membrane oxygenation. Most were treated with antibiotics (26 [84%]) and corticosteroids (24 [77%]). Corticosteroid regimens were variable but tended to consist of 40 to 60 mg of prednisone daily with a duration ranging from a few days to 2 weeks. Slightly more than one-third of patients (11 [36%]) were discharged while still receiving supplemental oxygen.

**Table 5.** Treatment and Outcomes

Treatment or outcome	Patients, No. (%) (N = 31)^a^
Admitted	
Hospital	28 (90)
ICU	8 (26)
Hospital length of stay, median (IQR), d	4 (3-7)
Highest respiratory support	
Room air	4 (13)
Nasal cannula	19 (61)
High flow nasal cannula	5 (16)
Noninvasive positive pressure ventilation	0
Mechanical ventilation	2 (7)
Extracorporeal membranous oxygenation^b^	1 (3)
Treatment	
Steroids	24 (77)
IV methylprednisolone	16 (52)
Starting daily dose, median (IQR), mg^c^	60 (60-156)
Planned duration, median (IQR), d	7 (1-15)
Antibiotics	26 (84)
Pulmonary function tests at follow-up, median (IQR)	
Predicted FEV_1_, %	92 (83-104)
Predicted FVC, %	99 (93-107)
FEV_1_/FVC	84 (75-86)
Predicted DLCO, %	76 (64-83)

Abbreviations: DLCO, diffusing capacity for carbon monoxide; FEV_1_, forced expiratory volume in 1 second; FVC, forced vital capacity; ICU, intensive care unit; IQR, interquartile range; IV, intravenous.

^a^ Percentages may not sum to 100 due to rounding.

^b^ One patient was supported with venovenous extracorporeal membranous oxygenation.

^c^ Doses expressed in milligram equivalents of prednisone.

### Readmissions

Two patients (6%) were rehospitalized after discharge. One (3%) was not initially treated with corticosteroids after a single dose of prednisone induced a manic reaction. After discharge, he resumed vaping THC and was readmitted with new lung opacities, a new subpleural cystic space in the right middle lobe, a contiguous pneumothorax, and pneumomediastinum, the last attributed to the Macklin phenomenon. He improved with chest tube drainage and reinstitution of corticosteroids. The other patient was discharged with a 5-day course of prednisone but did not complete it. He was rehospitalized 3 days later with worsening hypoxemia and progressive pulmonary opacities. Corticosteroids were reinstituted, and he improved with continued supportive care.

### Follow-up

All patients survived, and 20 patients (65%) were seen in follow-up. Median (IQR) time to follow-up after hospital discharge was 16 (11-28) days, and at the time of follow-up, no patients required supplemental oxygen. Ten patients (50%) had follow-up chest radiographs, which were either normal or demonstrated near complete resolution of opacities. Pulmonary function tests were performed in 18 patients (85%) and demonstrated normal spirometry (median [IQR] percentage of forced vital capacity exhaled in first second [FEV1/FVC], 83.5% [75.3%-86.0%], median [IQR] predicted FVC, 99.0% [93.3%-106.5%], and median [IQR] predicted FEV_1_, 92.0% [82.8%-103.8%]) but mildly reduced predicted diffusion capacity (median [IQR], 76% [64%-83%]). All patients improved, but residual symptoms were common, with 13 (65%) reporting shortness of breath, 9 (45%) reporting cough, and 2 (10%) reporting chest pain. Small numbers of patients continued to report nausea and vomiting (2 [10%]), fatigue (2 [10%]), night sweats (1 [5%]), and weight loss (1 [5%]). Seven patients (35%) had no residual symptoms at the time of follow-up.

## Discussion

We report a retrospective series of cases of EVALI, an emergent flu-like respiratory illness with the potential for high morbidity and mortality,^11^ seen at the University of Utah during the height of the epidemic in 2019. Our patients were evaluated and managed at an academic medical center using a relatively uniform and comprehensive diagnostic strategy with high rates of bronchoscopy and chest CT imaging as well as a substantial early follow-up rate. Thus, this series complements but extends data from previous reports and enhances our understanding of this novel disease.

Our findings confirm those of previous series by showing that EVALI presents as a flu-like illness with respiratory, constitutional, and gastrointestinal symptoms as well as elevated serum inflammatory markers. However, more patients in previous series were treated in an ICU and fewer underwent bronchoscopy. In the seminal series by Layden et al,^1^ 52 of 98 patients (53%) were cared for in an ICU, and 43 patients (44%) underwent bronchoscopy. In the series by Blagev et al,^12^ 33 of 60 patients (55%) were admitted to an ICU, and 19 patients (32%) underwent bronchoscopy. More recent additions to the literature, including those by Zou et al,^20^ Henzerling et al,^14^ and MacMurdo et al,^16^ demonstrated similar ICU admission rates (range, 46%-47%) and bronchoscopy rates (range, 19%-47%). By comparison, only 8 (26%) of our patients were treated in an ICU, and a much larger proportion of all patients (22 [77%]) underwent bronchoscopy. The reason for the lower rate of ICU admission in our series is not readily apparent but may reflect a lower severity of illness or different thresholds for ICU utilization. Of note, the demographic characteristics of our cohort are comparable to other series^1,12,14,15,16,20^ in that they are comprised largely of young, White men who frequently use THC products, with substantial psychiatric comorbidity rates. This argues against underlying demographic dissimilarities as the cause of lower ICU admissions.

There are several reasons for the high rate of bronchoscopy in our series. First, before EVALI became a well-recognized clinical entity, bronchoscopy was performed as part of a general diagnostic survey for infection, diffuse alveolar hemorrhage, acute eosinophilic pneumonia, and so on.^32,33,34,35^ Second, the consistent identification of LLMs in BAL specimens reinforced this practice. Finally, we used bronchoscopy as the most sensitive means of excluding alternative diagnoses and reducing diagnostic uncertainty given that EVALI was considered a diagnosis of exclusion.^33,36^

Similar to previous series and case reports, we found LLMs in most cytological samples from BAL fluid.^1,4,12,13,17^ LLMs are a nonspecific cytopathological finding that are observed in a raft of diseases including lipoid pneumonia, amiodarone toxicity, and aspiration.^22,25,37^ It is unknown whether LLMs are a marker of EVALI, a marker of vaping, or a marker of vaping products that contain the causative agent of EVALI.^13,17,23,24^ Future studies are needed to determine whether LLMs are useful in the diagnosis of EVALI. Our data show only that they are typically present.

Current clinical guidance is undecided regarding the role of bronchoscopy in the evaluation of patients with suspected or possible EVALI and emphasizes the role of clinical discretion in individual cases.^1,12,38,39^ Our data show that in suspected cases of EVALI during the apogee of the epidemic, bronchoscopy rarely contributed meaningfully to diagnosis. This suggests that in the absence of risk factors for or symptoms of a specific infectious or other alternative diagnosis, bronchoscopy is not routinely necessary, as has been argued by other authors.^16^ There were no complications of bronchoscopy in our patients, but a recent article reported a high rate of adverse events among younger patients, further arguing for limiting the use of bronchoscopy in typical cases of EVALI.^40^

Our finding of 3 false-positive PCR results for *Pneumocystis jirovecii* may be surprising to some readers. The most stringent, criterion standard for a diagnosis of *Pneumocystis pneumonia* incorporates longitudinal evaluation of clinical and radiographic data as well as response to treatment or lack thereof. Using that standard, the specificity of PCR testing for *Pneumocystis* is on the order of 80% to 90%,^41,42^ meaning that in persons without clinical *Pneumocystis* disease, 10% to 20% will have the organism detected by PCR due to colonization rather than infection. When a test with this level of specificity is used in a low prevalence population (such as ours, lacking risk factors for *Pneumocystis pneumonia*), most positive tests will be false-positives, as dictated by Bayes theorem. Failure to incorporate information regarding disease prevalence into diagnostic estimates has been termed base rate neglect,^43^ and it may go undetected in clinical practice if all positive PCR results are presumed to represent clinical illness and are treated for *Pneumocystis *pneumonia.^44^ Our series, by happenstance alone, buttresses these general principles of diagnostic reasoning because our patients were not treated and all improved, confirming that the PCR results were false-positives.

A wide array of patterns of injury on CT imaging have been reported in EVALI.^3,26,37,45,46^ Our series is among the first to document the variability of imaging patterns within a cohort. Most patients had the OP pattern as the exclusive finding, a feature witnessed to a high degree in other studies.^16^ The prototypical OP pattern comprises multifocal, multilobar, ground glass opacities, often contiguous with uninvolved pulmonary lobules, and sometimes with sparing of the peripheral, subpleural lung. We caution that this very prevalent imaging pattern does not predict pathologic findings in the lung. None of our patients had biopsies, but 1 report of 8 patients with EVALI showed OP on histopathological examination of biopsy specimens in just 4 cases (50%).^17^

All patients in our series had good outcomes, although nearly two-thirds had residual symptoms at follow-up. These symptoms as well as normal spirometry and abnormal diffusion capacity testing align with the findings in previous series.^12,15,20^ Because EVALI has substantial overlap with other causes of acute lung injury, it may have a similar natural history and result in residual respiratory morbidity on long-term follow-up.^47,48^ Follow-up CT imaging may be useful for reticular changes as well as quantitative evaluation of potential airway disease in patients with normal spirometry, as demonstrated in a large research study in obstructive lung disease.^27^ Longitudinal evaluation of symptoms and pulmonary function are also needed to better understand the natural history and long-term prognosis of EVALI.

### Limitations

This retrospective case series has all the limitations inherent in uncontrolled, observational data. Not all variables were present for every patient due to variations in care between clinicians. We may have missed cases of EVALI that were not brought to our attention or where the diagnosis of EVALI was missed. Some of our patients may have had pneumonia with negative diagnostic testing and improved with antibiotics and corticosteroids, so they were falsely identified as cases of EVALI. Our assessment of LLMs relied on a semiquantitative and nonstandardized assessment by the examining cytopathologist, intended for clinical use, not research. Our radiologists were not masked to the presumed diagnosis of EVALI when they jointly arrived at an informal consensus regarding radiographic patterns. Vaping histories were variable and difficult to quantify, as was administration of corticosteroids. Follow-up data were incomplete. In spite of these unavoidable limitations of observational research, this series adds meaningful data to the growing body of evidence regarding EVALI.

## Conclusions

EVALI is a serious flu-like respiratory syndrome that mimics infectious pneumonia. In this study, patients presented with a characteristic but nonspecific constellation of respiratory, constitutional, and gastrointestinal symptoms as well as elevated serum inflammatory markers. CT imaging typically revealed an organizing pneumonia pattern. Bronchoscopy typically revealed LLMs but had an unacceptably high rate of results that were not indicative of true infection. These observations may help physicians recognize prototypical cases of EVALI, make a provisional clinical diagnosis without invasive testing, and then assess response to abstinence from vaping with or without corticosteroid administration. Most patients improve with these measures; however, residual symptoms and abnormalities on pulmonary imaging and function are present in a significant proportion of patients at early follow-up. Additional work is needed to determine the long-term prognosis of this novel and potentially deadly syndrome.

## Notes

Supplement. **eFigure 1. **CT Imaging in 5 EVALI Cases **eFigure 2. **Airway Wall Thickening in Right Upper Lobe in 2 Patients
