# Supplementary material for: Clinical, Bronchoscopic, and Imaging Findings of e-Cigarette, or Vaping, Product Use–Associated Lung Injury Among Patients Treated at an Academic Medical Center
Source: JAMA Netw Open. 2020 Nov 6;3(11):e2019176. doi: 10.1001/jamanetworkopen.2020.19176 (PMC7648253; doi:10.1001/jamanetworkopen.2020.19176)
Supplement: Supplement. — eFigure 1. CT Imaging in 5 EVALI Cases eFigure 2. Airway Wall Thickening in Right Upper Lobe in 2 Patients [file jamanetwopen-e2019176-s001.pdf]

Supplemental Online Content

Aberegg SK, Cirulis MM, Maddock SD, et al. Clinical, bronchoscopic, and imaging findings of e-cigarette, or vaping, product use–associated lung injury among patients treated at an academic medical center. *JAMA Netw Open*. 2020;3(11):e2019176. doi:10.1001/jamanetworkopen.2020.19176

eFigure 1. CT Imaging in 5 EVALI Cases
eFigure 2. Airway Wall Thickening in Right Upper Lobe in 2 Patients

This supplemental material has been provided by the authors to give readers additional information about their work.

© 2020 Aberegg SK et al. *JAMA Network Open.*

eFigure 1. CT Imaging in 5 EVALI Cases

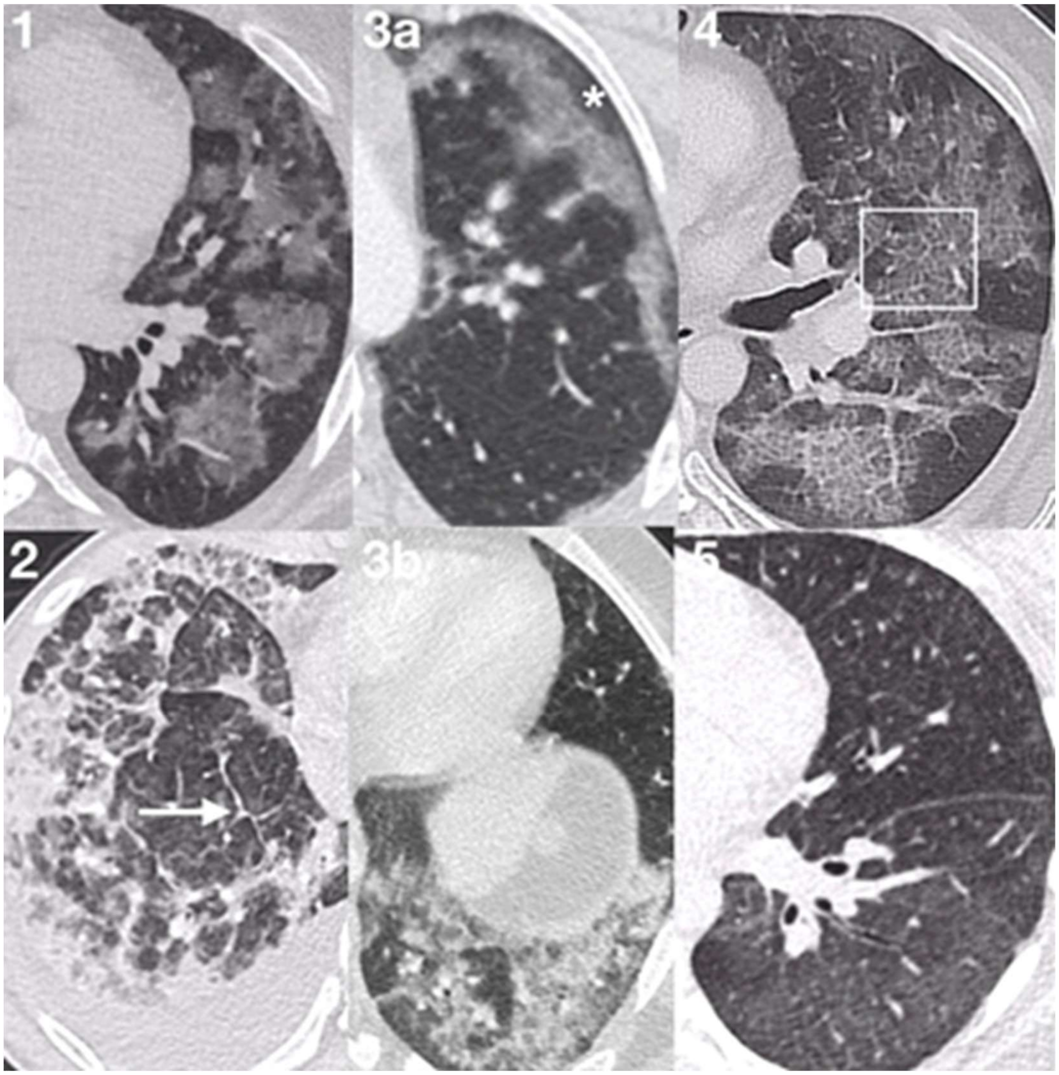

1. Prototypical OP pattern. 2. AEP pattern with septal lines (white arrow) and pleural fluid. 3a. OP pattern, with upper zone ground glass opacities and subpleural sparing *. & 3b. Consolidative opacities in lower lung zone. 4. ELP pattern with *crazing paving* (in white box). 5. HP pattern, with mild, diffuse ground glass opacity in lower lobe and subtle centrilobular nodules.

© 2020 Aberegg SK et al. *JAMA Network Open.*

eFigure 2. Airway Wall Thickening in Right Upper Lobe in 2 Patients

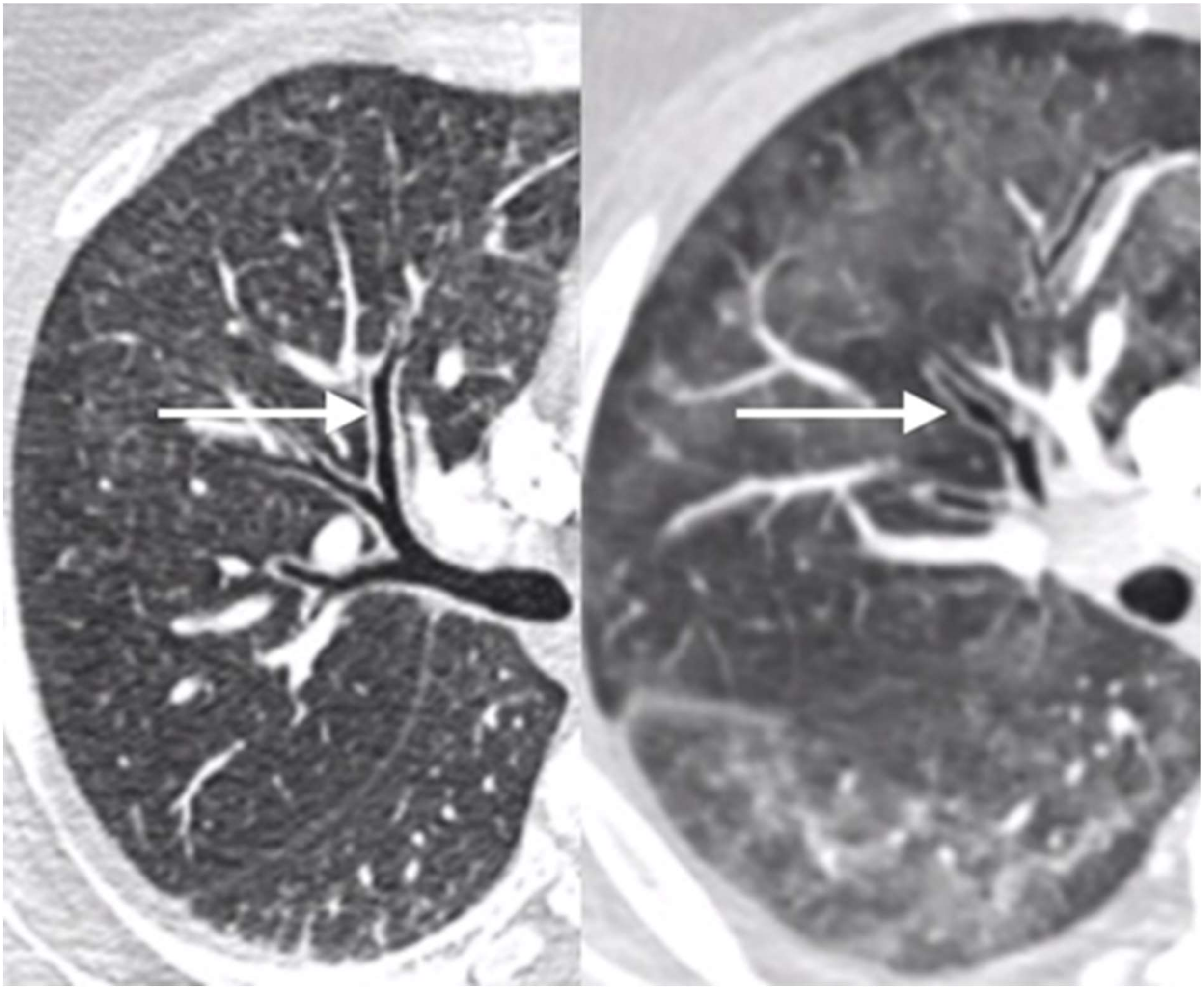

© 2020 Aberegg SK et al. *JAMA Network Open.*
